# Role of preliminary registry data in development of a clinical trial for an innovative device: a small but integral piece of a health policy initiative

**DOI:** 10.1080/20016689.2017.1283106

**Published:** 2017-03-02

**Authors:** Donald R. Ricci, Joost de Vries, Raphael Blanc

**Affiliations:** ^a^ Division of Cardiology, University of British Columbia, Vancouver, B.C., Canada; ^b^ Evasc Medical Systems Corporation, Vancouver, B.C., Canada; ^c^ Department of Neurosurgery, Radboud University Nijmegen, Nijmegen, Netherlands; ^d^ Assistant Chief of Interventional Radiology, Ophthalmological Foundation A. de Rothschild, Paris, France

**Keywords:** Clinical trial, intracranial bifurcation aneurysm, health policy, registry data, medical device

## Abstract

Establishing a national health policy at a macro level involves the integration of a series of health initiatives across a spectrum of activities, including clinical care. Evaluation of the safety and efficacy of a new medical device ultimately evolves to testing in humans. The pathway to a formal prospective clinical trial includes a stepwise appreciation of pre-clinical data and detailed analysis of data obtained from preliminary registries, where information about appropriate patient selection and use of the device is obtained. Evaluation of procedural and follow-up efficacy and safety data in a preliminary series of cases, chosen to simulate published data, allows the design and conduct of clinical trials that are required to verify preliminary observations, closing the loop on one aspect of modifying health policy decisions.

## Introduction

Establishing a national health policy at a macro level involves the integration of a series of health initiatives across a spectrum of activities, including clinical care. The World Health Organization’s statement is that ‘outcomes can be improved through increased and more focused investment in monitoring and evaluating how national health policies, strategies, and plans are implemented … when properly designed, this allows for learning, continuous improvement of the planning process and timely corrective measures. It also contributes to documenting policy reform processes.’[1] One of these activities is the conduct of a clinical trial designed to determine utility of a new drug or device in the management schema of a disease entity.

Evaluation of the safety and efficacy of a new medical device begins with intensive pre-clinical (‘bench’ and animal) testing but must, at some stage, evolve to testing in humans who suffer from the disease entity that the device was designed to treat. This should occur cautiously and deliberately by highly experienced and skillful clinicians familiar with the disease entity, the device, the clinical milieu and the conduct of clinical trials. The purpose of this brief communication is to describe the rationale used to initiate a formal prospective clinical trial of an innovative device, eCLIPs (eVasc Neurovascular Enterprises ULC, eVasc Medical Systems Corp, Vancouver, Canada), using data from an ad hoc registry developed for the purpose. The approach to training for the use of new medical devices has already been described.[2]

## Study perspective

**Figure 1.**
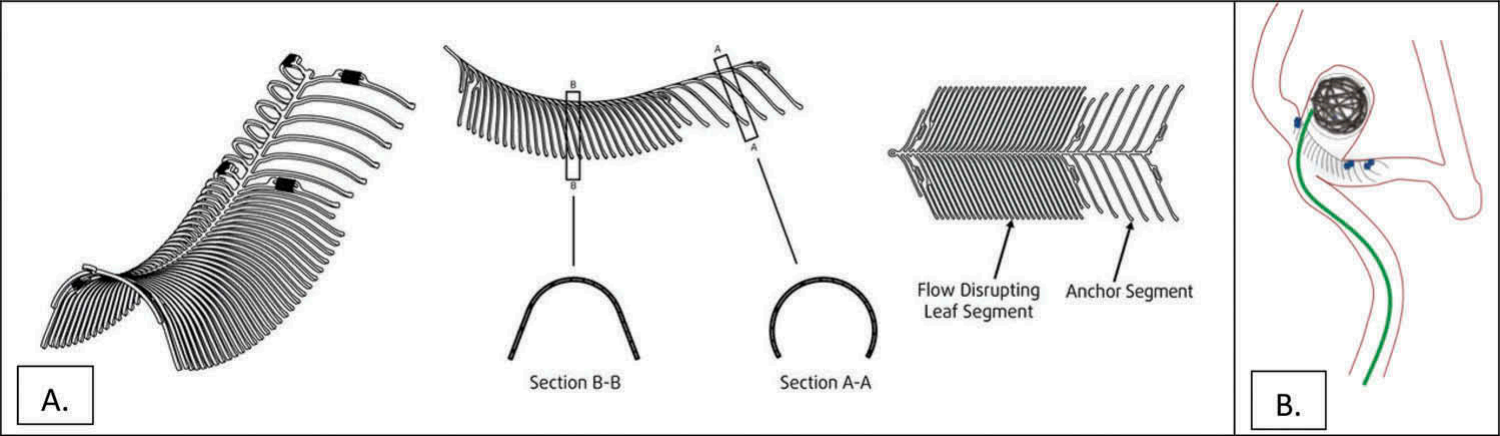
(a). Illustration of eCLIPs device in 3-D (left), side (middle) and plan (right) views. (b). Illustration of eCLIPs device deployed with anchor segment in sidebranch and leaf portion bridging the neck, behind which are coils delivered by catheter penetrating ribs of leaf portion.

The eCLIPs Device is a self-expanding nitinol non-circumferential device with anchor and leaf segments (Figure 1), the latter bridging the neck and allowing for coil retention, flow diversion, a platform for endothelial growth so that the aneurysm closes permanently by thrombosis and cicatrization, and allowing the device to be incorporated into the vessel wall.

It was designed to embody features that met specific unmet needs in management of intracranial bifurcation aneurysms, particularly those with a wide neck, an especially complex type of anatomy (i.e. the basilar artery or carotid terminus) that have had a variety of off-label treatment approaches that have not resulted in consistently good long term outcomes. The features of the eCLIPs system include: device removable, retractable and repositionable before detachment; non-shortening on deployment; absence of device migration; stable platform during coil delivery; coil retention; flow disruption away from the aneurysm; platform for endothelial growth; no compromise of access to side branches and good wall apposition accruing to its non-circumferential design. Pre-clinical clinical assessment of the eCLIPs device in a rabbit model of bifurcation aneurysms shows adherence to these features required for definitive and sustained aneurysm exclusion from the circulation and incorporation of the device into the vessel wall to produce a physiologic remodeling of the aneurysm.[3]

Un-ruptured or stabilized ruptured aneurysms at the bifurcation of the basilar artery or carotid terminus are a rare anatomic subset of bifurcation intracranial aneurysms. Currently no standard of care is available to manage this type of aneurysm. Current options for the endovascular treatment of bifurcation aneurysms include (i) simple coiling (no stent) and balloon remodeling; (ii) the use of commercially available stents ‘off-label’ to create a Y- or T-stent in conjunction with coils; (iii) coil retaining devices; and (iv) intrasaccular devices. However, all these techniques have limitations.

Simple coiling of bifurcation aneurysms is associated with significantly higher aneurysm recurrence rates compared to sidewall aneurysms: a recurrence rate of 35% at an average follow-up of 30 months,[4] a large proportion of which was due to coil compaction.[5]

Stent assisted coiling using a variety of stents and various techniques has not improved on the aneurysm recurrence rate at between 18% and 37%.[6–10]

**Figure 2.**
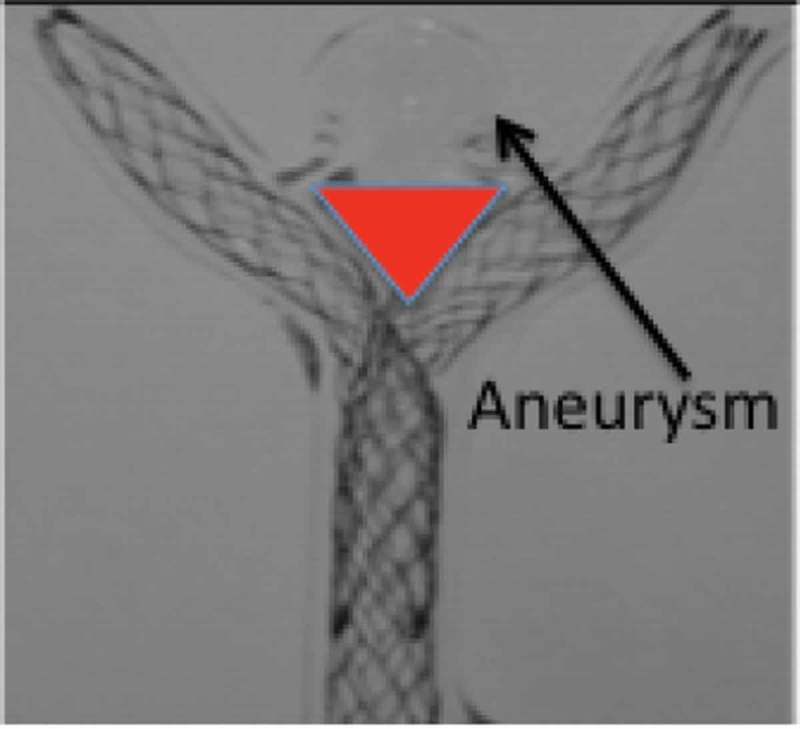
Illustration of dual Y-stenting in bifurcation aneurysm glass model, showing triangular gap between stents and neck of aneurysm.

Y-stent assisted coiling, first proposed by Chow et al in 2004,[11] is performed in Y-shaped bifurcations by placing two stents in the parent artery with each stent in one of the bifurcation branches, creating a new bifurcation point across the neck of the aneurysm.[11] However, placing two stents in this manner is a compromise that does not allow either stent to bridge the aneurysm neck at its mid-portion because of a necessary triangular gap that exists (Figure 2); it leaves more metal in the arterial system permanently to serve as a source for thrombi or emboli, impedes access to side branches and perforators, and doubles the cost compared with placing one stent in optimal position.[12] Small series have confirmed the feasibility of the procedure, but associated with high risk. Bartolini et al. [13] suggested that Y and X stent-assisted coiling was associated with a high rate of complications, 10% procedure-related permanent morbidity, and 1% mortality rate. Outcome data for coil retaining devices are sparse and indicate in various time periods of follow-up an important rate of aneurysm recurrence or persistence.[14–18] Intrasaccular devices are gaining in popularity, but these also show a significant rate of aneurysm recurrence or persistence.[14,19–23] The primary reason for the universal and apparently consistent rate of recurrence at the site of a bifurcation is speculated to be the unimpeded water hammer effect, or the jet effect of blood flow, from the main vessel into the aneurysm,[24,25] resulting in coil compaction. Coil retaining devices do not mitigate the water hammer effect. Compaction of intrasaccular devices is also apparent.[22] The features designed into the eCLIPs device, particularly the neck bridging and its attendant attributes, may reduce or obliterate this effect.

## Methodology

Initial eCLIPs implants took place in 2013 under Health Canada’s Special Access Program. This program ‘permits health care professionals to access custom-made and unlicensed medical devices for emergency use or when conventional therapies have failed, are unavailable or are unsuitable to provide a diagnosis, treatment or prevention for patients under their care.’[26] The intent of these implants was to obtain ‘first in human’ experience in a patient population that had no other therapeutic option and, at the discretion of their treating physicians, had a reasonable chance of clinical benefit from the eCLIPs device. Experience using the eCLIPs device also came from European implants after granting of CE Mark status in 2014. Each case, and its follow-up, was reviewed by a multidisciplinary team. This combined early experience, comprising a registry covering 13 international centers, allowed the operators to learn the nuances of device implantation – the ‘learning curve’[2] and identify anatomic variants that are particularly suited, and those that are not, to eCLIPs usage. Critical to this process is collection of acute procedural and follow-up safety and outcome data in all patients. These earliest results have been reported [27] and contain the entirety of the experience in the first 33 patients, including patients who would not qualify under CE Mark granted Indications for Use (‘treatment of intracranial aneurysms arising from bifurcation branch artery diameters in the range of 2.0 mm–3.25 mm’) or FDA Humanitarian Use Designation criteria (‘intracranial saccular aneurysm with a diameter of >5 mm arising at the internal carotid artery bifurcation or the basilar artery bifurcation, with a bifurcation branch artery diameter in the range of 2.0 mm–3.25 mm’). In order to obtain data that would allow at least an approximate comparison with published outcomes, it is important to analyze results in patients who meet certain inclusion and exclusion criteria. These results may then define a cohort that may be the subject of a formal prospective clinical trial.

## Results

### Efficacy

**Figure 3.**
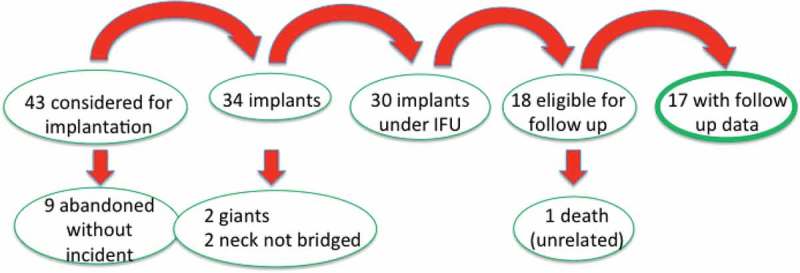
Graphic illustration of disposition of all patients considered for eCLIPs implantation from 2013 to 31 May 2016.

Figure 3 shows a flow chart of the entire clinical activity to 31 May 2016, and includes data already published (to September 2015).[27]

**Figure 4.**
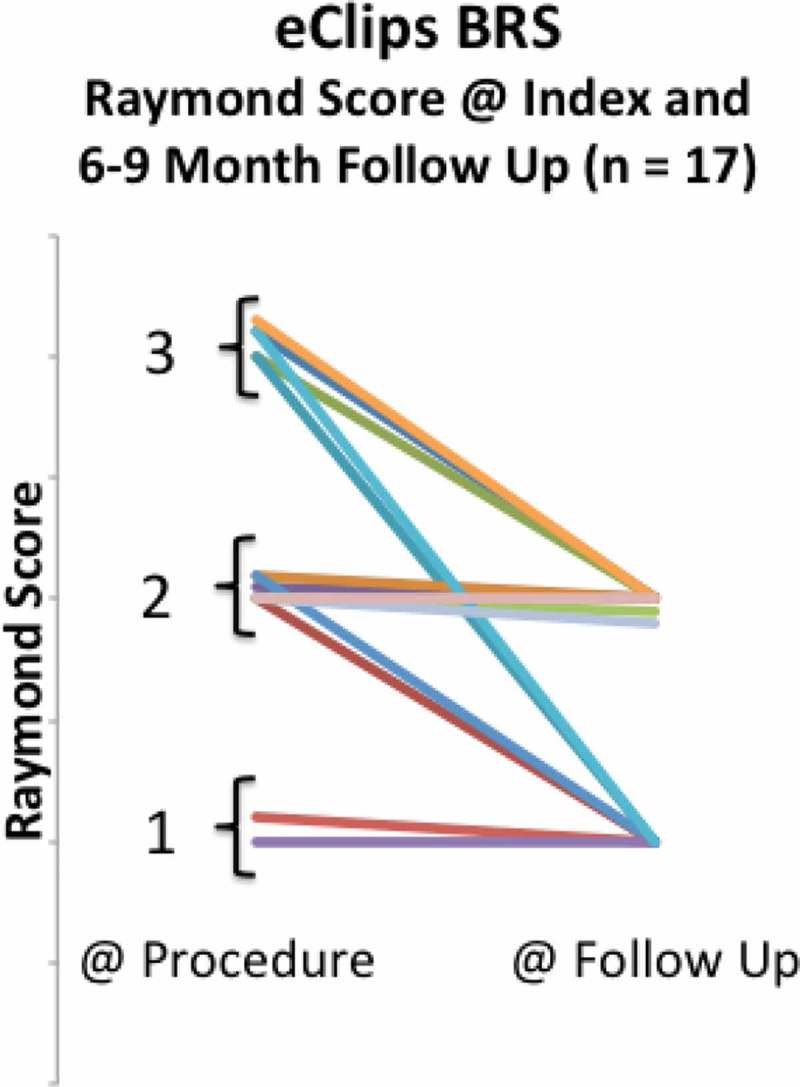
Raymond scores at index procedure and at follow-up in all patients reaching at least six-month anniversary after eCLIPs implantation.

As of May 2016, 43 patients were considered for eCLIPs implantation at a bifurcation, the majority at basilar and carotid termini. The eCLIPs Device was successfully deployed in 79% of patients considered for eCLIPs treatment (34/43). The reasons for non-deployment – the nine abandoned cases – included a change in or a difference in interpretation of the anatomy from the screening CTA to digital angiography at the time of the procedure, and caution in application of a new device in challenging anatomic situations (‘learning curve’ issues). Of the 34 implants, two of the patients did not meet inclusion criteria (giant aneurysms) and two with very wide and broad necks had a strategy of ‘neck narrowing’ rather than full bridging of the neck with the eCLIPs device. Therefore, 30 patients met criteria that ordinarily would be included in a clinical trial of bifurcation aneurysms, and comparative to most of the published literature for this anatomic subset. Of these, 18 reached at least a six-month time period after the index procedure and available for imaging and clinical follow-up. One of these died of an unrelated traumatic event,^1^ ^1^Traumatic, alcohol-related (Denmark) eight months after the procedure but before follow-up imaging could be completed, leaving 17 patients, presented in Figure 4.

From the Index procedure to follow-up, no patient had a regression in Raymond Score.[28]

**Figure 5.**
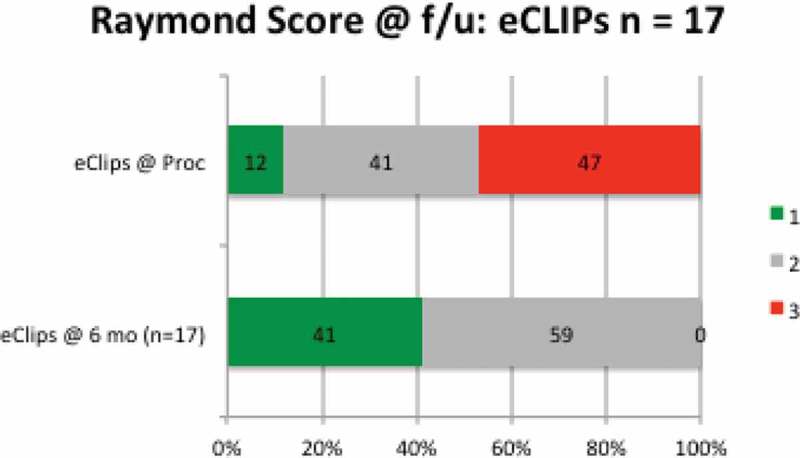
Percentage of patients allocated to Raymond scores 1–3 at index procedure and at follow-up.

Shown more analytically in Figure 5, at the time of the procedure, 12% (N = 2/17) of patients had achieved complete aneurysm occlusion (Raymond 1), 41% (N = 7/17) had partial occlusion (Raymond 2), and 47% (N = 8/17) had persistent aneurysms (Raymond 3), indicating loose coiling. At the 6–9-month follow-up, no patient had a persistent aneurysm (Raymond 3) (N = 0 /17), and all had either Raymond 1 score (41%) or Raymond 2 score (59%).

### Safety

Complications in the entire cohort of 43 patients are shown in Table 1.[27]

**Table 1.** Complications documented in all patients considered for implantation of eCLIPs device.

Complication	Number of patients	Comment
Death^2^	3	2 giant^3^;[27] 1 guide wire perforation^4^
Stroke	1	Guide wire perforation^5^
Transient ischemic attack	2	Transient cortical blindness; transient aphasia

**Table 2.** Procedural adverse events documented in all patients considered for implantation of eCLIPs device.

Adverse events	Number of patients	Comment
Dissection	1	Asymptomatic^6^
Vasospasm	4	Asymptomatic; resolved with catheter removal and administration of vasodilating agent^7^
Thrombotic event	2	Asymptomatic^8^

^2^In eCLIPS implanted patients,^3^ two (N = 2/34) adverse events (Table 2) ^4^occurred after the eCLIPS procedure.^5^ There was one instance of residual at the neck region which was recoiled three months later. There was another instance where a patient^6^ had symptoms of subarachnoid hemorrhage (SAH)^7^ one week after the eCLIPs procedure requiring re-treatment with six nano-coils.[27]^2^No deaths occurred at the time of eCLIPs procedure
^3^Late rupture and mass effect; death at 10 and four months, respectively (Canada)
^4^Hemorrhage over the pericallosal region, with mass effect inferiorly; subsequent review of case images suggested that the bleeding was likely due to distal wire perforation during first branch access (UK).
^5^Distal to the target site (i.e. not device related); amongst the group in whom no eCLIPs device was implanted.


## Discussion

**Figure 6.**
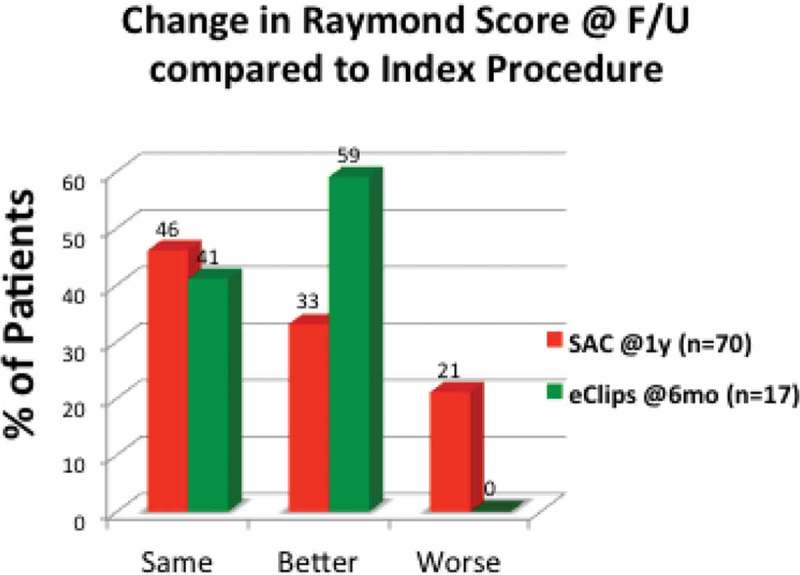
Comparative follow-up data versus Index procedure (eCLIPs vs. stent assisted coiling, SAC).

^8^This continued improvement of Raymond Score from procedure to follow-up in patients treated with eCLIPs as seen in Figure 3 is unique compared to current methods used to treat bifurcation aneurysms, most notably stent assisted coiling (SAC), as illustrated in Figure 6.

Typically, SAC is associated with regression in at least 20% of cases from procedure to follow up.[7] ^6^Caused by initial 4 F French diagnostic catheterization catheter (not eCLIPs catheters) requiring stenting.
^7^Two of these were related to placement of the eCLIPs microcatheter, a common occurrence with catheter-based treatments in the cerebral vasculature, and in one case resulted in the decision not to deploy the device.
^8^One secondary to antiplatelet therapy resistance, resolving spontaneously with removal of the device; second involved platelet aggregation of thrombus after eCLIPs deployment requiring microcatheter targeted abciximab administration.


**Figure 7.**
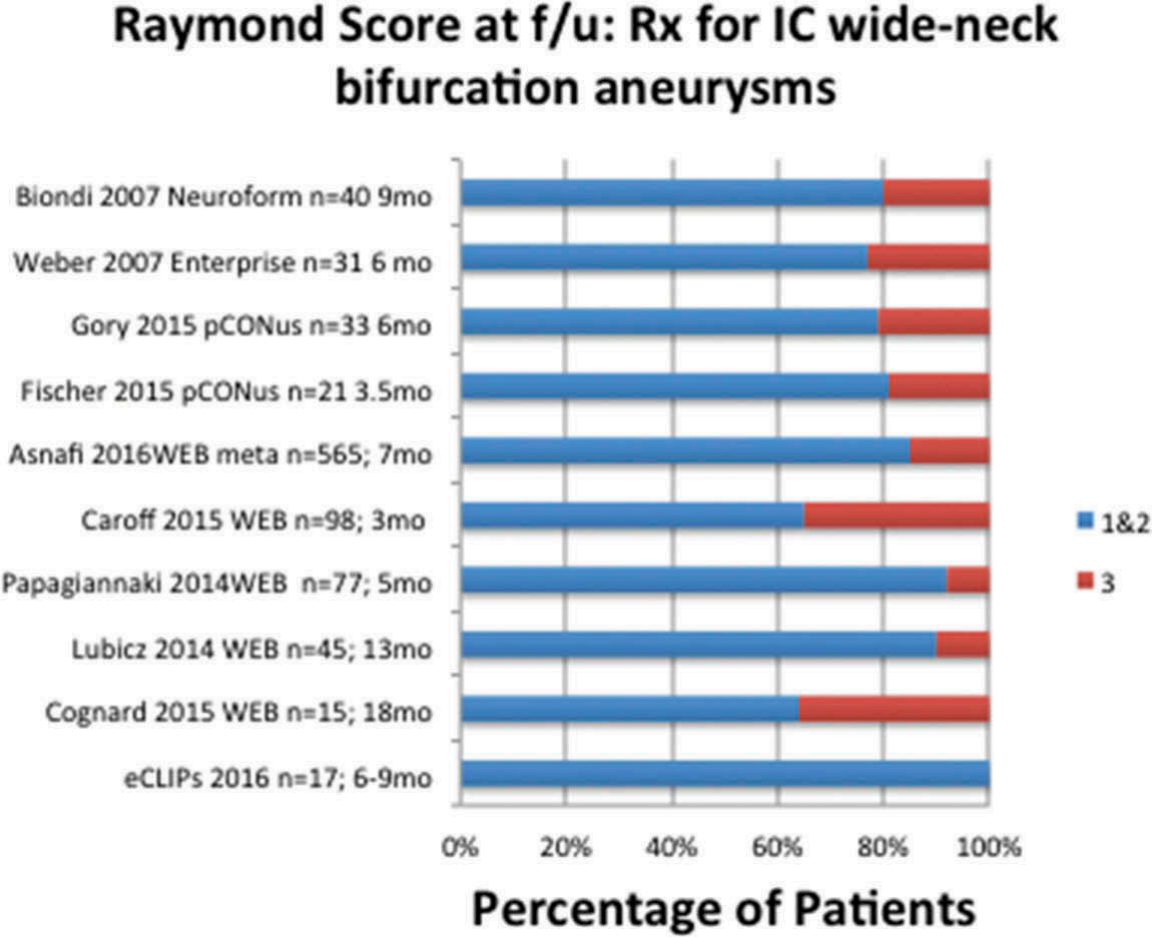
Percentage of patients allocated to Raymond Scores 1–2 and 3 in spectrum of studies of devices used to manage wide-neck bifurcation aneurysms.

The efficacy results of the preliminary eCLIPs registry of patients meeting inclusion criteria for management of wide-neck bifurcation aneurysms, though numbers are small, show superior results to preliminary results from clinical trials with similar inclusion criteria for alternative management options for bifurcation aneurysms, illustrated in Figure 7. While no eCLIPs patient had a Raymond 3 score at follow-up, patients treated with a variety of devices reportedly have 20–37% Raymond 3 at follow-up.

In other words, across the gamut of devices used to manage this complex anatomic subset, dual stenting, coil-retaining devices, and intrasaccular devices, persistent or recurrent aneurysms (Raymond Score 3) are common at follow-up.

The safety profile is analogous to that reported for similar trials.

## Conclusion

The preliminary efficacy results from the on-going registry support the potential clinical benefit of eCLIPs in patients with bifurcations aneurysms, particularly at the basilar and carotid termini. In this population, these data highlight successful access to the target vasculature and deployment of eCLIPs device with an acceptable safety profile. The registry suggests an unprecedented long-term improvement to the patient outcome of treating bifurcation intracranial aneurysms with a favorable aneurysmal occlusion rate and no evidence of recurrence or persistence at six months follow-up.

These results are favorable enough to justify a formal prospective clinical trial for verification.

A thoughtful and transparent analysis of preliminary registry data can identify a patient cohort with similar characteristics as exists in published reports, as justification for developing a formal prospective clinical trial whose outcome should be integral to furthering clinical care policies and treatment paradigms, particularly in rare and complex conditions.
